# Covalent Warheads Targeting Cysteine Residue: The Promising Approach in Drug Development

**DOI:** 10.3390/molecules27227728

**Published:** 2022-11-10

**Authors:** Fangjiao Huang, Xiaoli Han, Xiaohui Xiao, Jinming Zhou

**Affiliations:** 1 Key Laboratory of the Ministry of Education for Advanced Catalysis Materials, Department of Chemistry, Zhejiang Normal University, 688 Yingbin Road, Jinhua 321004, China; 2 Drug Development and Innovation Center, College of Chemistry and Life Sciences, Zhejiang Normal University, 688 Yingbin Road, Jinhua 321004, China

**Keywords:** cysteine, covalent warheads, covalent inhibitor

## Abstract

Cysteine is one of the least abundant amino acids in proteins of many organisms, which plays a crucial role in catalysis, signal transduction, and redox regulation of gene expression. The thiol group of cysteine possesses the ability to perform nucleophilic and redox-active functions that are not feasible for other natural amino acids. Cysteine is the most common covalent amino acid residue and has been shown to react with a variety of warheads, especially Michael receptors. These unique properties have led to widespread interest in this nucleophile, leading to the development of a variety of cysteine-targeting warheads with different chemical compositions. Herein, we summarized the various covalent warheads targeting cysteine residue and their application in drug development.

## 1. Introduction

Covalent inhibitors are a class of small molecule compounds that can covalently bind to specific target proteins, thereby inhibiting their biological functions. For a long time, the off-target side effects of covalently bound drugs have been a substantial problem, which limits their potential for drug development [1]. Nonetheless, it is beginning to be recognized that focusing on a specific protein and the mechanism of action, not just the molecular target, but also often the specific binding site and desired mode of action must be specified at the beginning of the project. More and more covalent drugs have been reported and even successfully applied in clinical, such as the proton pump inhibitor omeprazole, the anticoagulant clopidogrel, and the non-small-cell lung cancer inhibitor afatinib, which made people re-recognize the potential of covalent drugs.

Compared with non-covalent inhibitors, one of the most important advantages of covalent inhibitors is their high binding affinity to target proteins, which may result in a relatively long duration of action [2]. In addition, covalent bond inhibitors can also reduce drug resistance caused by target mutations. For example, MRTX849 (adagrasib), developed by Mirati Therapeutics, has been identified as a highly selective covalent inhibitor of KRAS (G12C) and is currently in phase I/II clinical studies. It is an oral selective inhibitor of small molecule KRAS (G12C) mutations. It not only inhibits KRAS mutation almost completely in vivo, but also exhibits favorable drug-like properties [3]. Currently, cysteine is the most common covalent amino acid residue in a variety of covalent drugs, and various warheads have been developed that can react with cysteine, providing the key building blocks for covalent drugs to form covalent bonds. In this review, we summarized the various covalent warheads targeting cysteine residue and their application in the development of cysteine-based covalent drugs.

## 2. Covalent Inhibitors

**Figure 1:**
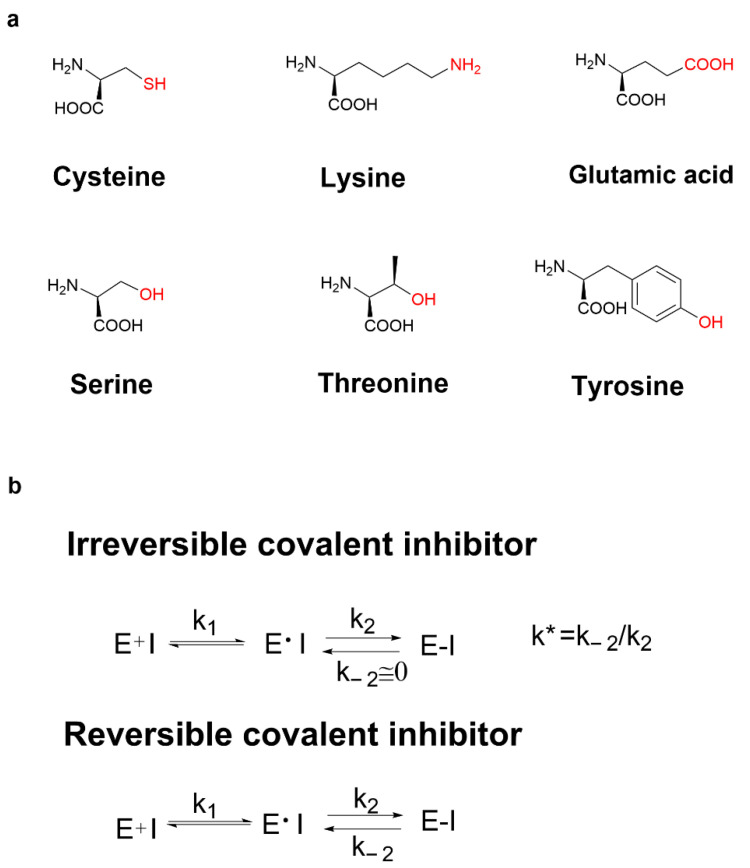
(**a**) The covalent reaction-related residues; (**b**) mechanism action of irreversible covalent inhibitors and reversible covalent inhibitors.

Recently, various covalent drugs have been emerging. The development of covalent drugs contains many fields such as anticancer, antiviral, and diabetes. In general, covalent binding is much more stable than non-covalent binding. Therefore, compared with classical non-covalent inhibitors, covalent inhibitors have many potential benefits such as prolonging the duration of action, improving ligand efficiency, avoiding drug resistance when targeting amino acids required for enzyme catalysis, and targeting the non-conserved amino acids in high selectivity [4]. Moreover, small-molecule modulators that selectively bind to target proteins can be served not only as tools for understanding protein function but also as clues for drug discovery. While most small molecules function by interacting with their biological targets under equilibrium binding conditions, ligands that chemically modify proteins through the covalent bond formation can offer potential advantages, including improving ligand potency, prolonging the duration of action, enhancing the ability to overcome resistance mutations, and facilitating the identification of target proteins [5]. It has been reported that the covalent reaction-related residues in proteins can be cysteine, lysine [6,7], glutamic acid [8], serine [9,10], threonine [11] and tyrosine [12], etc. (Figure 1a). However, the most common covalently reactive residue is the poorly conserved non-catalytic cysteine [13].

Unlike non-covalent inhibitors, covalent inhibitors block their target proteins in two steps. Covalent binding of covalent inhibitors to target proteins first requires the formation of target protein–inhibitor complexes through non-covalent binding, which is similar to the binding mechanism of non-covalent inhibitors via an equilibrium process. In the second rate-determining step, the electrophilic warhead of the covalent inhibitor is appropriately positioned near amino acid residues at the binding site. Irreversible covalent inhibitors and reversible covalent inhibitors are also different. Compared with the first step, the rate of covalent bond formation is relatively slow, and there is a reaction equilibrium constant k* if the rate constant of the reverse reaction for the dissociation of the inhibitor–protein complex (k_−2_) is equal to or close to zero, that is to say, when k_2_ is much larger than k_−2_, k* tends to infinity, then the inhibitor is called an irreversible covalent inhibitor, such as clopidogrel (Figure 1b). When the difference between k_2_ and k_−2_ is not very large, that is, when k* is in a reasonable range, it can be called reversible covalent binding. The electrophilic warheads of reversible covalent inhibitors are mostly reversible nucleophilic addition reaction receptors such as cyano- and keto-carbonyl groups, such as sitagliptin (Figure 1b). 

## 3. Covalent Inhibitors Covalently Bound to Cysteine

### 3.1. Cysteine Profile

**Figure 2:**
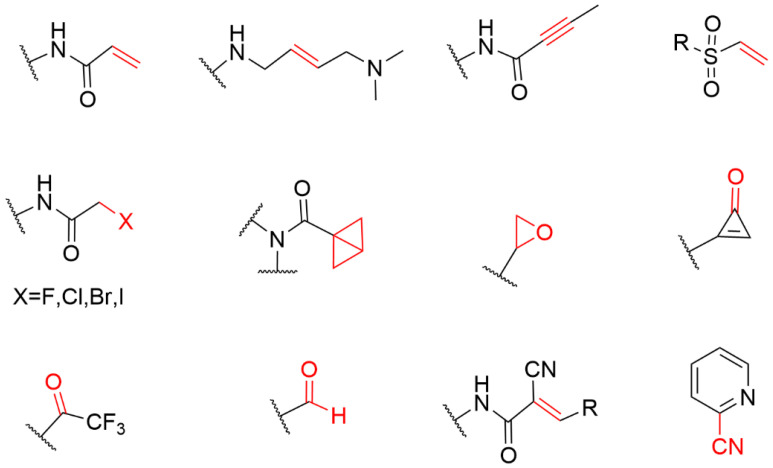
The general structure of the electrophilic library sorted by warhead chemistries.

Cysteine is one of the least abundant amino acids in proteins of many organisms, which plays a crucial role in the catalysis, signal transduction, and redox regulation of gene expression [14]. Due to the large atomic radius of sulfur and the low dissociation energy of the S-H bond, the thiol group of cysteine possesses the ability to perform nucleophilic and redox-active functions that are not feasible for other natural amino acids. The thioether group in methionine and the sulfhydryl group in neutral amino acids are only moderately nucleophilic; however, once it exists in the cysteine side chain as a thiolate, its nucleophilicity increases by several orders of magnitude, thus becoming the most nucleophilic nucleophile among the 20 common amino acids. Therefore, cysteine has become the most common covalent amino acid residue in covalent drug development [15]. cysteines are very rare, and they have been shown to react with a variety of warheads, especially Michael receptors [16,17]. These unique properties have led to widespread interest in the nucleophile, leading to the development of a variety of cysteine-targeting warheads with different chemical compositions (Figure 2) [18].

### 3.2. Cysteine-Directed Covalent Drugs

The potency and selectivity of small-molecule drugs can be improved via introducing cysteine-targeting elements that can covalently bind to their targets [13,19]. Most covalent inhibitors have been designed to target the highly nucleophilic thiol groups of cysteine residues [20]. However, the irreversible nature of these covalent inhibitors might increase the severity of off-target effects, and further optimization of covalent drugs is always required [21]. Recently, there has been mounting interest in developing irreversible inhibitors that can form covalent bonds with cysteines or other nucleophilic residues in the ATP-binding pocket. Gray et al. described the distribution of accessible cysteines and divided cysteines into five groups spanning the entire kinase to facilitate the design of covalent inhibitors. Cysteine residues located in different parts of the binding sites of various targets provided potential opportunities to develop specific irreversible inhibitors using different cysteine positions [22]. Much work in the field of covalent drug development has focused on targeting cysteines with Michael receptors. In particular, acrylamide-based inhibitors have achieved great success. For example, the U.S. Food and Drug Administration (FDA) has approved the marketing of the anticancer drug ibrutinib which covalently inhibited Bruton’s tyrosine kinase (BTK) [23]. Ibrutinib formed irreversible covalent bonds with cysteines near the active site of BTK and has been successfully used to treat B-cell carcinomas [24]. In addition, Gray et al. also reported a selective covalent fibroblast growth factor receptor (FGFR) inhibitor targeting covalent cysteines located at various positions within the ATP-binding pocket, which was of great interest for overcoming the most common resistance to kinase inhibitors [25]. The cyclin-dependent kinase 7 (CDK7) inhibitor THZ1 covalently binds irreversibly to Cys312 of CDK7 located near the kinase domain via the acrylamide moiety [26]. Therefore, both reversible and irreversible inhibitors have the potential to target cysteine. Reversible covalent cysteine-directed drugs have the potential advantage that they are less likely to exhibit off-target effects and instead form covalent adducts with cysteines, thereby increasing specificity and possibly reducing toxicity [27].

## 4. The Covalent Warheads Targeting Cysteine Residue

The electrophilic functional groups on covalent inhibitors are called “warheads” and form covalent bonds with nucleophilic residues such as the cysteines on the target protein. Following, we summarized various warheads of cysteine covalently bound drugs.

### 4.1. Heteroaromatic Warheads

While nucleophilic aromatic substitution (S_N_Ar) reactions have a long history in covalent protein targeting, heteroaromatic electrophiles have received relatively little attention. In this type of reaction, covalent labeling is achieved via the replacement of a leaving group from an electron-deficient aromatic ring by a nucleophile, such as a cysteine thiol (acid salt). That is, the S_N_Ar reaction proceeds through a stepwise addition-elimination mechanism [15].

**Figure 3:**
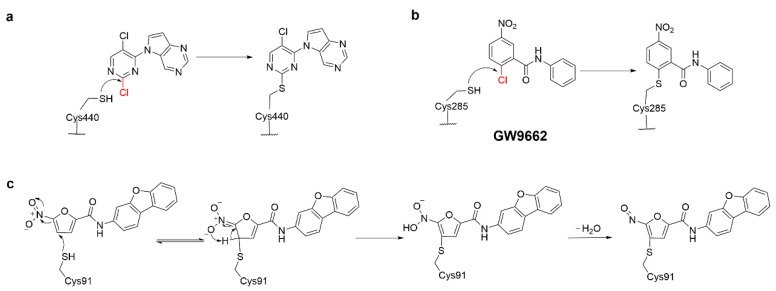
The mechanism of covalent nucleophilic aromatic substitution (**a**) 2, 5-dichloropyrimidine with cysteine, (**b**) GW9662 with cysteine, and (**c**) mechanism of the reaction of C-178 with Cys91.

In early applications, S_N_Ar warheads have been used as covalent inhibitors. Jason Yano et al. identified a covalent inhibitor of MSK1 C-terminal kinase domain (CTKD), in which 2,5-dichloropyrimidine underwent S_N_Ar reaction with Cys440 (Figure 3a) [28]. In addition, *p*-chloronitrobenzene (GW9662) has been identified as a cysteine-responsive peroxisome proliferator-activated receptor (PPARγ) with a nanomolar IC_50_ against PPARγ (Figure 3b) [29].

Ablasser’s group screened and identified a nitrofuran derivative C-178, which blocked STING activation-induced palmitoylation by covalently targeting Cys91, thereby preventing STING from assembling into a multimeric complex in the Golgi apparatus and inhibiting its downstream signaling. The possible mechanism of covalent bond formation between C-178 and Cys91 was shown in Figure 3c. Further biological assay indicated that C-178 effectively and selectively inhibited the STING response elicited by different true activators [30]. The analog of C-178, H-151, exhibited great potential in the treatment of autoimmune diseases including amyotrophic lateral sclerosis, psoriasis, etc.

**Figure 4:**
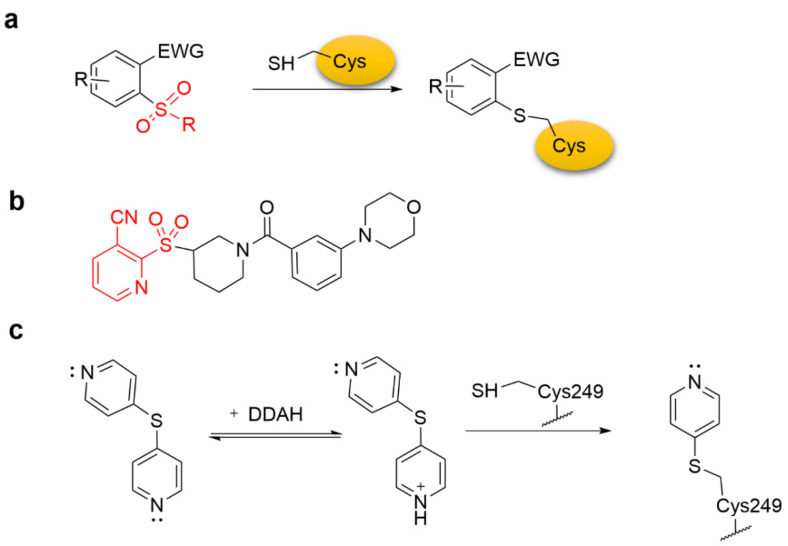
(**a**) The mechanism of 2-sulfonylpyridine forming a covalent adduct with cysteine. (**b**) Chemical structure of 2-sulfonylpyridine-based irreversible covalent ADA modifier. (**c**) 4-Pyridylsulfide undergoes S_N_Ar reaction with active site Cys249.

Zambaldo et al. reported a series of 2-sulfonylpyridines as tunable S_N_Ar-based reactive groups that selectively reacted with bio thiols via nucleophilic aromatic substitution (Figure 4a). They demonstrated the potential of the 2-sulfonylpyridine reactive group by discovering a selective covalent modulator of adenosine deaminase (ADA) (Figure 4b) [31]. Moreover, 4-pyridylsulfide also underwent the S_N_Ar reaction with Cys249 at the active site of dimethylarginine dimethylaminohydrolase (DDAH) (Figure 4c) [32]. 

### 4.2. α,β-Unsaturated Carbonyl Warhead

Currently, targeting non-catalytic cysteine residues with α, β-unsaturated carbonyl compounds is the main strategy to develop targeted covalent inhibitors (TCIs), which typically utilizes the acrylamide group as a Michael acceptor because it is weakly electrophilic and requires proximity to cysteine residues to form covalent bonds, minimizing the interaction with another cellular thiol group to produce the off-target reactions [33].

#### 4.2.1. Acrylamide Warhead

**Figure 5:**
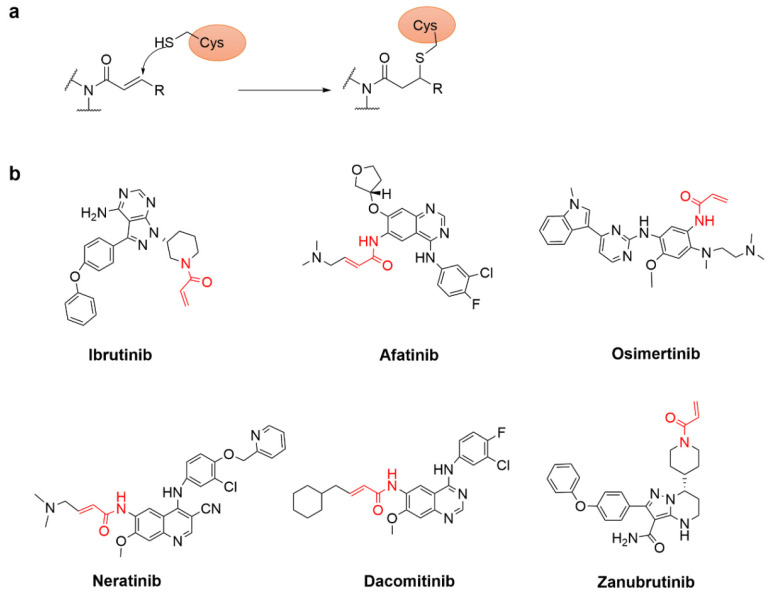
(**a**) The mechanism of Michael’s addition of acrylamide warheads to cysteine. (**b**) Small molecule covalent inhibitors with acrylamide warheads.

Targeting non-catalytic cysteine residues with irreversible acrylamide inhibitors is an effective approach to enhance pharmacological potency and selectivity [34]. The alkene moieties of acrylamide warheads react irreversibly with the thiol group of cysteine via Michael addition and form conjugated adducts (Figure 5a) [35]. The following are some irreversible covalent inhibitors targeting cysteine for Michael addition (Figure 5b). The oral inhibitor ibrutinib was approved for marketing in 2013 as a drug for the treatment of B-cell malignancy, in which nucleophilic cysteine residues near the pocket were interacted covalently by the Michael addition of the acrylamide group with a sub-nanomolar inhibitory activity against BTK [36]. Because the EGFR non-covalent inhibitor was prone to generate drug resistance during the treatment of cancer patients, the second-generation EGFR inhibitor afatinib was active at nanomolar levels in lung cancer cells through the covalent attachment to Cys797 of the mutant EGFR [37]. However, the T790M mutation reduced the affinity of afatinib for the covalent attachment to Cys797, which may lead to clinical toxicity and lack of efficacy [38]. Later, AstraZeneca developed the third-generation EGFR irreversible inhibitor osimertinib which could selectively target EGFR containing T790M mutation. Furthermore, the following neratinib [39] and dacomitinib [40] also exhibited good potency against T790M EGFR [17]. In 2019, the FDA approved the marketed drug zanubrutinib which is covalently bound to a cysteine residue in the active site of BTK to inhibit BTK activity [41]. In summary, acrylamide forms a covalent bond via Michael addition to cysteine.

#### 4.2.2. α-Cyan Acrylamide Warhead

**Figure 6:**
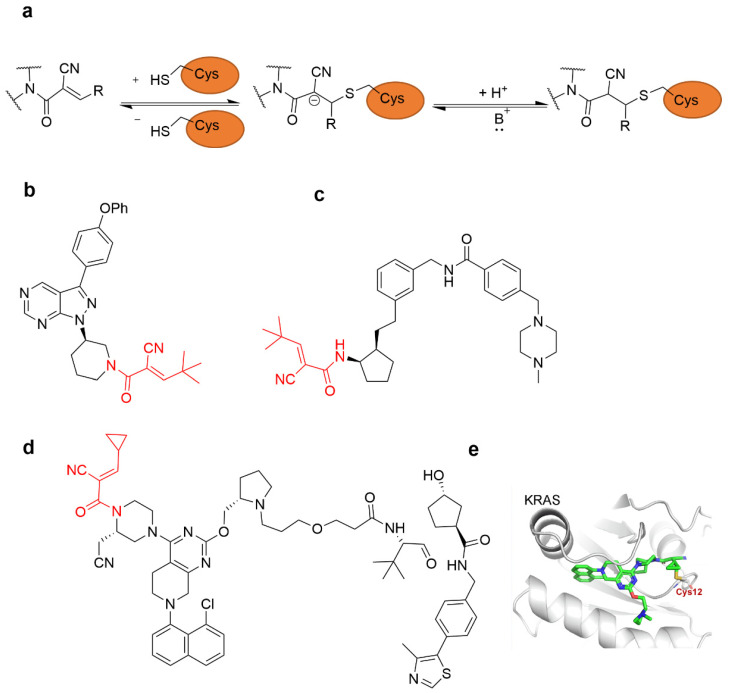
α, β-Unsaturated carbonyl moiety cyanoacrylamide as cysteine-targeting covalent warheads. (**a**) Mechanism of cyanoacrylamide forming a reversible covalent adduct with cysteine. (**b**) A cyanoacrylamide-based reversible covalent BTK inhibitor. (**c**) A cyanoacrylamide-based PPARγ phosphorylation inhibitor. (**d**) The chemical structure of designed PROTAC YF135. (**e**) The predicted binding mode of 6d (red) with KRAS^G12C^.

The acrylamide will appear as nucleophilic attack-related off-target phenomenon. Therefore, to avoid the off-target effect of acrylamide covalent inhibitor, the introduction of an electron-withdrawing cyan group at the α position accelerates the Michael addition to cysteine thiols and forms a reversible covalent bond (Figure 6a) [42]. Taunton et al. were the first to propose a reversible covalent inhibitor of the synthetic p90 ribosomal protein S6 kinase RSK2 by introducing an electron-withdrawing cyan group at the α-position of acrylamide [34]. At present, they have developed a series of reversible covalent BTK inhibitors and synthesized a series of reverse cyan acrylamide electrophiles using pyrazolopyrimidine scaffolds linked to ibrutinib (Figure 6b) [2]. The introduction of a cyan group at the α-position of acrylamide can not only increase its electrophilicity and its reactivity with cysteine residues but also greatly enhance the acidity of α-H, so the reverse reaction of Michael addition can biologically occur in vivo. On the other hand, by adjusting the steric hindrance of the β group, the removal rate of α-H can be regulated, thereby modulating the rate of the reverse reaction of Michael addition. The greater the steric hindrance of the β group, the more difficult the α-H on the drug molecule in the enzymatic pocket is to be removed by the base, and the lower the rate of the reverse reaction of Michael addition will be, thus increasing the action time of the drug [2]. Moreover, in 2019, Park et al. applied a cyanoacrylamide warhead to a PPARγ phosphorylation inhibitor that induced a reversible covalent bond with Cys285 of the PPARγ LBD (Figure 6c) [43]. Liu et al. reported a reversible covalent PROTAC YF135 based on cyanoacrylamide, which could form a reversible covalent bond with Cys12 of Kirsten rat sarcoma viral oncogene (KRAS)^G12C^ (Figure 6d,e) [44].

#### 4.2.3. Other Alkenes or Alkynes

**Figure 7:**
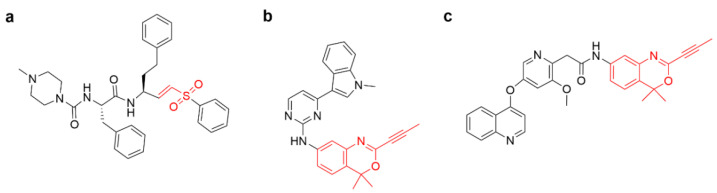
(**a**) Chemical structure of cysteine protease inhibitors. (**b**) Alkynyl benzoxazine-based JAK3 covalent inhibitor. (**c**) Alkynyl benzoxazine-based c-KIT covalent inhibitor.

In addition to the Michael addition reaction of acrylamide with thiols, other alkenes or alkynes can also undergo Michael addition reactions with thiols. In recent decades, vinyl sulfones have attracted much attention due to their potential for drug discovery and development [45]. For example, a compound is in the late stages of preclinical development for the treatment of Trypanosoma infection by potently and irreversibly inhibiting cysteine proteases (Figure 7a) [46,47]. More recently, McAulay et al. identified alkynyl benzoxazines and dihydroquinazolines as new warheads capable of covalently binding to cysteine. A potent covalent inhibitor with alkynyl benzoxazine warhead of JAK3 kinase (Figure 7b) potently improved the kinase selectivity and in vitro pharmacokinetic profile (Figure 7b) [48]. Furthermore, the cysteine of the receptor tyrosine kinase c-KIT could also be targeted by the alkynyl benzoxazine moiety (Figure 7c) [35].

### 4.3. Strain Release Motif Warhead

**Figure 8:**
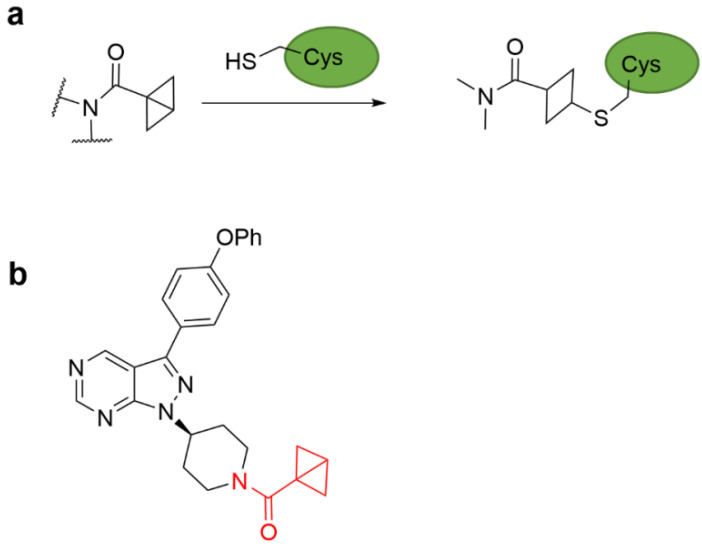
(**a**) Chemical structure of the BCB amide. (**b**) Chemical structure of the BCB amide-based irreversible covalent BTK inhibitor.

Among the strain-releasing motifs, the less studied bicyclo [1.1.0] butane (BCB) derivatives are used as electrophiles. BCB is stable under aqueous conditions and folds into a butterfly shape through bridged carbon–carbon bonds. The bridgehead carbon can undergo nucleophilic addition to the nucleophile cysteine thiol to open the ring (Figure 8a). This reactive feature of BCB amides has been successfully used to develop covalent ligands against BTK [49], which have higher selectivity than the covalent inhibitor with acrylamide-based warheads (Figure 8b). In 2017, Baran et al. proposed BCB sulfones as cysteine-directed warheads for chemo-selective covalent labeling of bioconjugates and target peptides [50]. Additionally, strain-driven nucleophilic addition of BCB amides can selectively and covalently target cysteine residues in living cells.

### 4.4. Alkyl Halide Warhead

**Figure 9:**
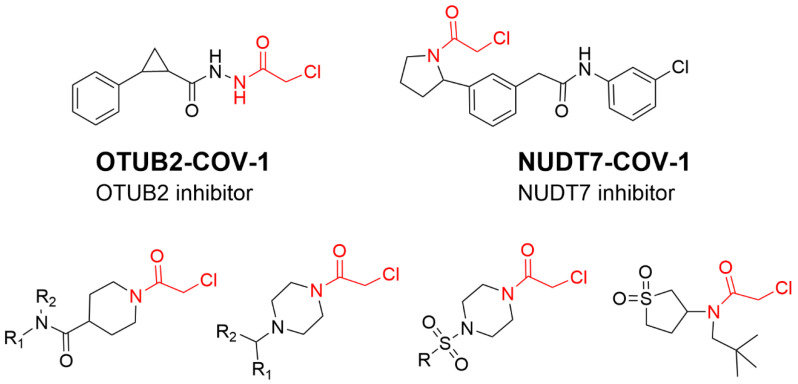
Chemical structure of representative chloroacetamide covalent fragment scaffolds targeting reactive cysteine.

Aryl halide warheads are reported to be relatively abundant in approved and investigational drugs, but their alkyl compounds are rare except for DNA alkylating agents [51]. α-Halo acetamides are another well-defined class of cysteine-directed electrophiles that react with thiols via the S_N_2 mechanism [52]. α-Halo acetamides are versatile and can be used to label various nucleophiles. For example, α-chloroacetamide is highly reactive with thiols under physiological aqueous conditions, which has been widely used in fragment-based drug discovery (FBDD) (Figure 9) [53]. For example, OTUB2-COV-1 targets the active Cys51 of OTUB2, and NUDT7-COV-1 targets C73 of NUDT7 [54]. Representative covalent fragment scaffolds target the active Cys145 of SARS-CoV-2 main protease (Mpro) [55]. Covalent inhibitors containing chloroacetamide warheads have been shown biologically active in vivo [56,57]. In 2021, Gray and London’s research group found a selective covalent inhibitor which could be used as an inhibitor of peptidyl-prolyl isomerase (Pin1) through targeting the Pin1 active site Cys113 (Figure 9). In a neuroblastoma-containing mouse model, oral administration of sulfadiazine induced down-regulation of the master regulator of gene transcription (c-Myc) targeted genes and regressed neuroblastoma in mice [58]. Importantly, the modification of chloroacetamide at the α position can alter its reactivity of cysteine thiols [59].

**Figure 10:**
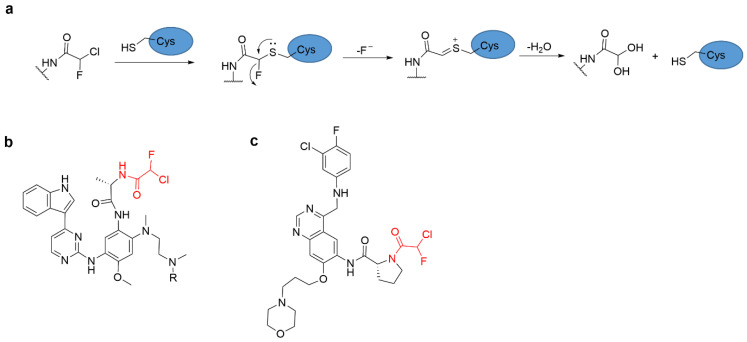
(**a**) The mechanism of the reaction of CFA with cysteine. Chemical structures of (**b**) α-chlorofluoroacetamide-based covalent inhibitor of EGFR (**c**) α-chlorofluoroacetamide-based covalent inhibitor NS-062.

Alpha-chlorofluoroacetamide (CFA) is a novel warhead for TCIs, which can form a covalent bond with a cysteine residue (Figure 10a). Despite weak intrinsic reactivity, CFA showed high reactivity to Cys797 of EGFR after the addition of quinazoline, and CFA-quinazoline showed higher EGFR targeting specificity than the corresponding Michael receptor in a certain concentration range (Figure 10b) [60,61]. Oral CFA-based EGFR inhibitor NS-062 (Figure 10c) inhibited tumor growth in mouse xenografts that targeted cysteine Cys797. 

**Figure 11:**
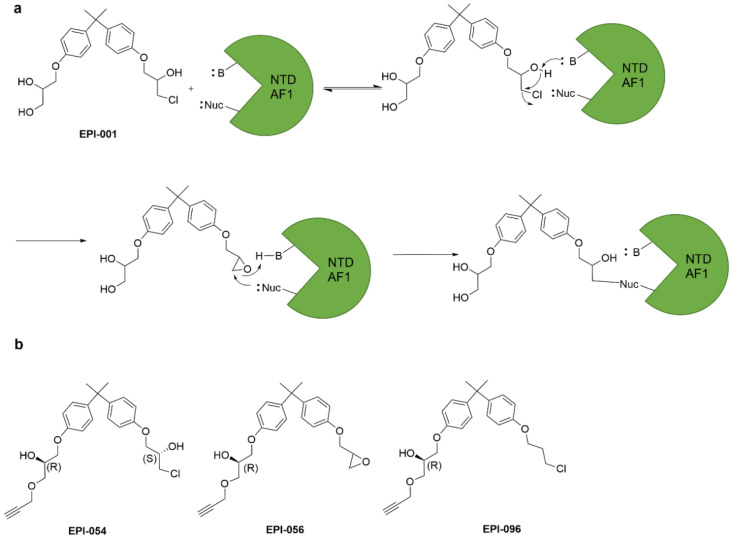
(**a**) Covalent binding reaction of EPI-001 compound to AR AF-1 region. (**b**) Chemical structures of EPI analogs.

EPI-001, an androgen receptor (AR) antagonist-like prostate cancer treatment drug developed by Essa Pharma (Figure 11a), can block the transactivation of the AR *N*-terminal domain (AR-NTD). The chlorine atom in the compound interacts with cysteine in the activation function-1 (AF-1) region to undergo an irreversible reaction to form a covalent bond, thereby inhibiting protein–protein interaction (AF-1) region in the NTD [62] and reducing AR interaction with androgen response elements on target genes. In LNCaP cells, EPI-001 inhibited androgen-dependent and androgen-independent cell proliferation, thereby inhibiting prostate cancer cell growth [62,63]. EPI analogs such as EPI-054, EPI-056, and EPI-096 (Figure 11b) overcame some of the limitations of current castration-resistant prostate cancer (CRPC) therapies, including EPI’s low propensity for gain-of-function mutations due to intrinsic disturbances in NTD and covalent binding. Importantly, EPI analogs are the only known inhibitors of constitutively active AR splice variants associated with CRPC, poor prognosis, and resistance to Abiraterone. The drug development paradigm can be applied to other intrinsically disordered proteins (IDPs) associated with cancer and other diseases [62].

### 4.5. Aldehyde Ketone Warhead

**Figure 12:**
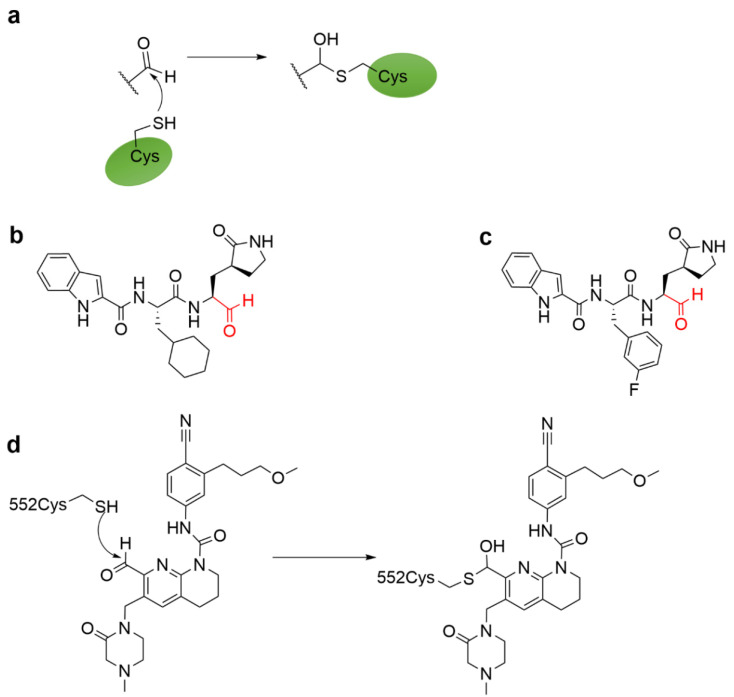
(**a**) The mechanism for the reaction of aldehyde groups with cysteine residues. (**b**,**c**) Chemical structures of the SARS-CoV-2 major protease (Mpro) inhibitors. (**d**) The mechanism of covalent adduct formation of FGFR inhibitors (red) with cysteine 552.

Aldehydes and ketones are more common warheads among proteolytic enzyme inhibitors [9,64,65]. Aldehyde and ketone-attached peptidomimetics act as reversible covalent inhibitors of cysteine proteases by preventing tetrahedral transition states by forming Hemi (thio) acetal and ketal complexes [66]. The mechanism for the reaction of aldehyde groups with cysteine residues is shown in Figure 12a. DAI et al. synthesized two inhibitors Figure 12b,c, which both strongly inhibited the activity of the main protease (Mpro) of SARS-CoV-2 and exhibited good anti-SARS-CoV-2 activity in cell culture. Both compounds formed a covalent bond with Cys145 of Mpro (Figure 12b,c), and showed good pharmacokinetic properties in vivo [67]. The clinical candidate compound 12d was obtained through the optimization of a series of lead compounds, in which the aldehyde in this compound underwent a reversible covalent Hemi thioacetal interaction with Cys552 (Figure 12d) [68]. It had the advantages of good oral bioavailability, pharmacokinetics (PK), and safety.

**Figure 13:**
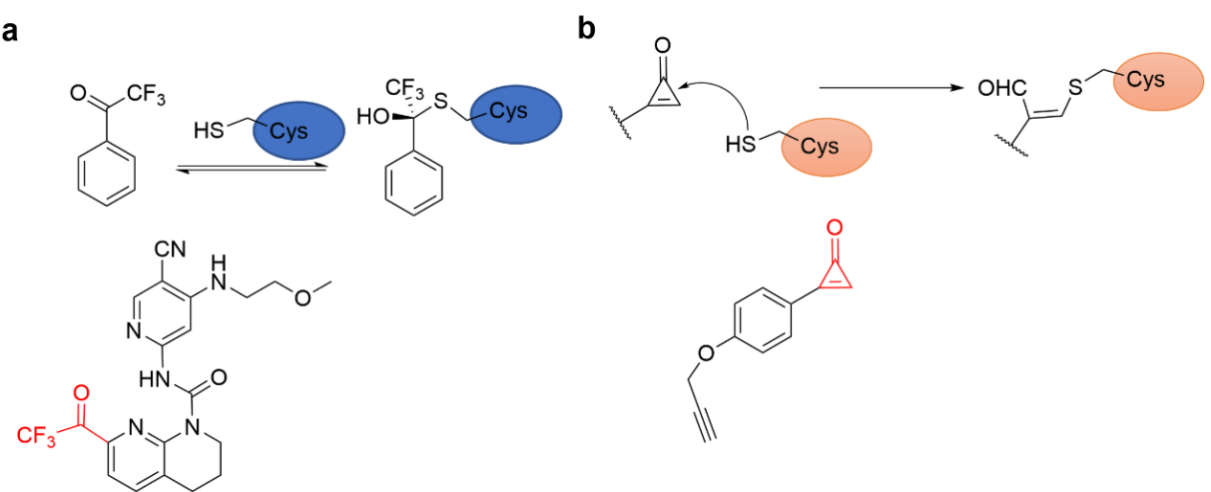
(**a**) The mechanism of reversible covalent adduct formation of aromatic trifluoromethyl ketones with cysteine residues and the chemical structure of an aryl trifluoromethyl ketone-based covalent reversible inhibitor of FGFR4 kinase. (**b**) The reaction mechanism of cyclopropenone with thiol groups and GSTP1 inhibitor with cyclopropenone warhead.

Ding’s group developed a new warhead, aromatic trifluoromethyl ketone used for covalent reversible kinase inhibitor design to target non-catalytic cysteine residues (Figure 13a). A potent and selective covalent reversible inhibitor of FGFR4 kinase was successfully designed and synthesized using this novel warhead. This functional group was also successfully applied to discover new JAK3 inhibitors (Figure 13a), suggesting its potential application in designing other kinase inhibitors [33].

Cyclopropenone is a powerful electrophile (Figure 13b) and has the potential to develop novel covalent inhibitors (Figure 13b). Cyclopropenone compounds inhibit the activity of glutathione S-transferase pi-1 (GSTP1) which is one of the triple-negative breast cancer drivers. The probe exhibited potent inhibitory activity against GSTP1 protein by binding to the catalytic Cys47 site. So, the cyclopropenone warhead can be served as a valuable warhead for developing potent GSTP1 inhibitors in cancer therapy [69].

### 4.6. Epoxides and Other Three-Membered Rings

**Figure 14:**
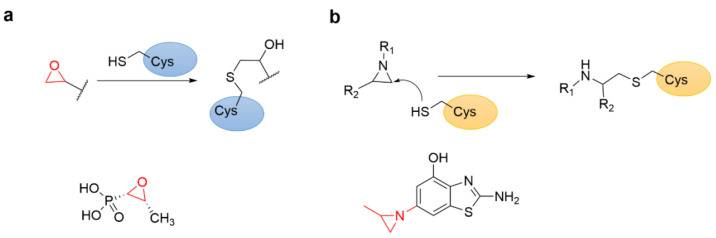
(**a**) The mechanism of covalent adduct formation between epoxides and cysteine and the chemical structure of fosfomycin. (**b**) The mechanism of aziridine forming a covalent adduct with cysteine and the chemical structure of aziridine-based irreversible covalent NSD1 inhibitor.

The epoxy group is often considered a warhead against different types of proteases in drug discovery since this group is widely used in the development of drugs and agrochemicals [70]. Compared with epoxides, other three-membered heterocycles such as aziridine [71] received relatively little attention. The epoxide reacts with the nucleophile through the S_N_2 mechanism to further open the ring (Figure 14a). In several epoxy compounds with medicinal value, the epoxy functional group forms a covalent bond with the target protein through a Cys residue [72]. For example, fosfomycin, isolated from Streptomyces (Figure 14a) [73] is marketed as an antibiotic for the treatment of urinary tract infections [74] and irreversibly inhibits N-acetylglucosamine enol pyruvate transferase (MurA) through the covalent interaction of its epoxy group with the key residue Cys115 for the catalytic activity of the enzyme [75,76]. 

Aziridine has also been developed as a cysteine-targeting warhead. The moiety has been studied and applied as mechanism-based inhibitor of glycosidase and cysteine protease (Figure 14b). Recently, Cierpicki et al. developed an irreversible covalent inhibitor with an aziridine moiety against nuclear receptor-binding SET domain protein 1 (NSD1) of histone methyltransferase (Figure 14b) [77]. The study demonstrated that the aziridine covalent warhead could target Cys2062 embedded in the hydrophobic site of the autoinhibitory loop [35].

### 4.7. Covalent Inhibitors Targeting Cyan Groups

**Figure 15:**
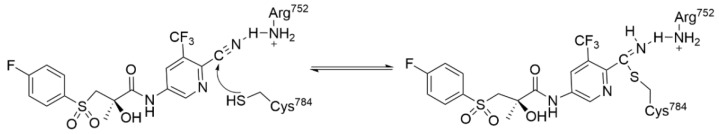
The reaction mechanism of 5N-bicalutamide with Cys784.

Nitriles have now been found to bind proteins or DNA in an irreversible manner, which has been identified as useful warheads for the inhibition of cysteine proteases. Importantly, unlike many electrophilic motifs, nitriles show good drug metabolism and pharmacokinetics (DMPK) properties. 5N-bicalutamide obtained by substituting the methine (CH) unit in the ortho-position of aryl nitrile of bicalutamide with nitrogen (N) atom, has significantly improved K_i_ compared to the parent compound and inhibitory activity against androgen receptor by forming a covalent adduct with Cys784 to prevent the current clinical anti-androgen resistance (Figure 15) [78].

**Figure 16:**
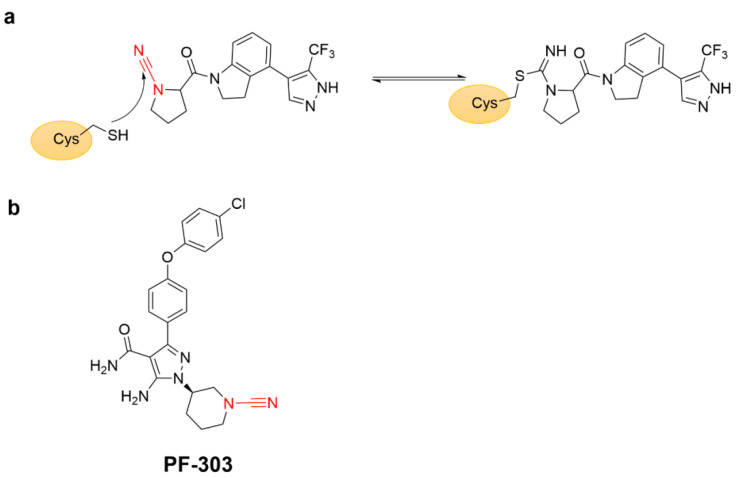
(**a**) The mechanism of covalent adduct formation between cyanopyrrolidine covalent inhibitor (red) and Cys481. (**b**) Chemical structure of the BTK inhibitor PF-303.

Particularly, nitriles have a long history as covalent reversible warheads for protease inhibitors [65]. Cyanamide is a representative of such type of reversible covalent warhead, and cyanopyrrolidine has been used to develop the inhibitor of cysteine protease (Figure 16a), especially for cathepsins and deubiquitinases [79,80]. Moreover, Micah Benson and colleagues from Pfizer identified an orally bioavailable BTK inhibitor PF-303 using cyanamide as a covalent warhead (Figure 16b). The cyanamide moiety in PF-303 formed a reversibly covalent bond with BTKs by modifying Cys481 near the ATP-binding pocket [81].

## 5. Conclusions

In this brief review, we introduce several warheads for covalent inhibitors, whose reaction types include nucleophilic substitutions (S_N_), Michael type (Ad_NM_), and non-Michael type (Ad_N_) type nucleophilic additions. The S_N_ types contain heteroaromatics, haloalkyls, epoxides, and other three-membered rings. The Ad_NM_ types contain the acrylamide, vinyl, and alkynyl groups mentioned in the review. WhiandAd_N_ types contain bicyclo [1.1.0] butane, aldehyde ketone, and cyano groups. Some of these covalent warheads have been thoroughly studied, validated, and even used in clinical therapy. During the optimization of cysteine targeting covalent inhibitors, we should first consider the optimization of the reactive warhead to adjust for the reactivity of the target cysteines and then use established fragmentation methods to optimize the non-covalent interactions. Although most recently approved TCIs use acrylamide Michael receptor-type warheads due to irreversible covalent inhibitors containing acrylamide-Michael receptor-type warheads, there is a certain risk of off-target toxicity and immune hypersensitivity reactions; based on this concept, many researchers have developed other new types of covalent warheads. Therefore, the clinical application of the recently developed electrophiles will be highly anticipated in the future.

Achieving high selectivity for off-target reactions requires that the intrinsic reactivity of the electrophilic warhead to the inhibitor must be low, and the reactivity of the selected covalent warhead must be the one that rarely forms reactive metabolites to reduce off-target interactions and reduce non-mechanical toxicity. The high specificity and potency of the inhibitor translate into lower and less frequent dosing, reducing the potential for off-target effects [82]. Furthermore, reversible covalent warheads represent another promising strategy to achieve refined target selectivity while the mitigating side effects caused by unwanted off-target reactions. Overall, the use of covalent inhibitor-based strategies is a reliable and rational approach to confer this important property.

## Data Availability

Not applicable.
